# Colon Cancer Pharmacogenetics: A Narrative Review

**DOI:** 10.3390/pharmacy10040095

**Published:** 2022-08-05

**Authors:** Álvaro Esteban Alfaro Alfaro, Brayan Murillo Castillo, Eugenia Cordero García, Javier Tascón, Ana I. Morales

**Affiliations:** 1 Faculty of Pharmacy, University of Costa Rica, San José 2060, Costa Rica; 2 Toxicology Unit, Universidad de Salamanca, 37007 Salamanca, Spain

**Keywords:** colon cancer, pharmacogenetics, pharmacological treatment, drug resistance, RAS, RAF, DPDY, DPD, TSYM, MTHFR, ABCB1, ABCB2

## Abstract

Currently, metastatic colon cancer is treated with monotherapeutic regimens such as folinic acid, fluorouracil, and oxaliplatin (FOLFOX), capecitabine and oxaliplatin (CapeOX), and leucovorin, fluorouracil, and irinotecan hydrochloride (FOLFIRI). Other treatments include biological therapies and immunotherapy with drugs such as bevacizumab, panitumumab, cetuximab, and pembrolizumab. After the research, it was found that some mutations make those treatments not as effective in all patients. In this bibliographic review, we investigated the pharmacogenetic explanations for how mutations in the genes coding for rat sarcoma virus (RAS) and rapidly accelerated fibrosarcoma (RAF) reduce the effectiveness of these treatments and allow the continued proliferation of tumors. Furthermore, we note that patients with mutations in the dihydropyrimidine dehydrogenase (DPDY) gene usually require lower doses of therapies such as 5-fluorouracyl (5-FU) and capecitabine to avoid severe adverse effects. Some other mutations in the thymidylate synthase gene (TSYM), methylenetetrahydrofolate reductase gene (MTHFR), and ATP binding cassette transporter B (ABCB1 and ABCB2) affect efficacy and security of the treatments. It is important to address the clinical implication of the oncologist in the study of gene mutations than can influence in the antitumoral response and safety of colon cancer treatments.

## 1. Introduction

The colon is an organ in the digestive system that extends from the ileocecal junction to the anus and is generally divided into six sections: the cecum, ascending colon, transverse colon, descending colon, sigmoid colon, and rectum [1,2]. It absorbs sodium ions, chloride, water (about 90%), and fatty acid chains and secretes potassium, bicarbonate, and mucus. This organ performs various movements to carry the intestinal contents to the anus. Its luminal surface contains epithelial cells in contact with the mucosa; this is surrounded by a layer of circular and longitudinal muscles, followed by the serosa and the visceral peritoneum [3]. The colon is characterized by a wide diversity of microorganisms that constitute the intestinal microbiota. This organ can suffer from several diseases, including colon cancer [4].

Colon cancer is a commonly diagnosed disease in both men and women [5]. According to the 2018 Global Cancer Observatory (GLOBOCAN) report based on GLOBOCAN data, colorectal cancer represented 10.2% of diagnosed tumors and 9.2% of deaths that year [6]. In Costa Rica, according to the same report, it was the third-most common tumor type (8.7% of cases) for both sexes, behind prostate cancer and breast cancer, which ranked first and second, respectively. In men, it was the second-most diagnosed tumor (8.6% of cases) behind prostate cancer. In women, it was the third-most common tumor type (8.8% of cases), surpassed by breast cancer (the most common) and thyroid cancer [7]. 

There are different kinds of colon cancer based on the tumoral cell’s origin from the embryological development. The most common kind of colon cancer is adenocarcinoma, which develops in the cells of the lining inside the large intestine. Cancers that are less common include primary colorectal lymphomas, gastrointestinal stromal tumors, leiomyosarcomas, carcinoid tumors, and melanomas [8].

Colon cancer has a substantial impact on public health, so research into this disease is essential. Our aim in this study was to describe the current treatments for metastatic adenocarcinoma colon cancer and the impact of various mutations on their efficacy and safety. To conduct this review, we searched several databases, including ResearchGate, the library, documentation, and information system—University of Costa Rica (SIBDI UCR), Google Scholar, and PubMed, and consulted GLOBOCAN and The National Comprehensive Cancer Network (NCCN) treatment guidelines, clinical trial reports, and other documents for information published in the last years (2010–2022). 

## 2. Colon Cancer Overview

### 2.1. Signs and Symptoms

One of the most important signs of the development of colon cancer is the formation of polyps in the intestine. The several types of polyps include adenomatous polyps (adenomas), hyperplastic polyps, inflammatory polyps, sessile serrated polyps (SSPs), and traditional serrated adenomas (TSAs) [9,10]. Other signs and symptoms may include bowel habit changes, maroon-colored or black stool, rectal bleeding, abdominal discomfort, abdomen fullness feeling, fatigue, and weight loss [8]. 

### 2.2. Risk Factors

The risk factors of colon cancer include age; a diet rich in red meat and low in fruits and vegetables [11,12]; obesity and physical inactivity [13]; smoking; alcohol consumption; microbiota composition [14,15]; type-2 diabetes; irritable bowel syndrome (IBS); relatives with colon cancer or polyps [16,17]; and inflammatory bowel disease (IBD), including Crohn’s disease (CD) and ulcerative colitis (UC). Some data suggest that IBD increases the risk of developing colon cancer by 0.5% to 1.0% annually [18,19].

Among the causal theories for this cancer, a genetic contribution has been established. The gradual accumulation of oncogene mutations may lead to the autonomous overproduction of colonic epithelial cells. These mutations slowly progress over a period of 10 to 40 years and lead to the first signs of colon adenomas, which can then develop into organ cancer. Differences exist between tumors in the right (ascending) colon and those in the left (descending) colon, with a worse prognosis in patients who develop the former. These differences are due in part to differential tumor gene expression [20,21,22,23,24].

### 2.3. Pharmacological Therapy

The drugs used in the pharmacological therapy of colon cancer include chemotherapy and biological drugs. This section describes the main treatments and their adverse effects.

#### 2.3.1. Chemotherapy

According to the NCCN guidelines, the clinical treatment of metastatic or advanced colon cancer begins with FOLFOX, CapeOX, or FOLFIRI, as shown in Table 1 [25,26].

The action mechanisms of the chemotherapy drugs used to treat colon cancer are explained below.

Oxaliplatin: This drug contains a platinum (Pt) atom that interacts with the nitrogen 7 of guanine in DNA and causes the formation of covalent adducts between two guanines, which interrupt DNA replication and transcription, resulting in cytotoxicity. Resistance to this therapeutic group needs to be investigated [27].

5-FU and capecitabine: These are both antimetabolites of the fluoropyrimidine group that indirectly interact with the folate cycle, inhibiting the enzyme thymidylate synthetase and thus the formation of deoxythymidine monophosphate (dTMP) and the production of thymidine. Capecitabine is an oral prodrug that is metabolized in the body to 5-FU. It should be used with leucovorin as a rescue therapy or concurrently with 5-FU as a treatment booster to reduce adverse effects [28,29].

Irinotecan: This drug is an analog of camptothecin. It is a prodrug that produces the metabolite SN-38, which inhibits the enzyme topoisomerase I. It causes the supercoiling of DNA, and it inhibits cell proliferation in processes such as translation, transcription, and mitosis [30].

#### 2.3.2. Biological Drugs and Immunotherapy

The mechanisms of cancer that can be attenuated using biological therapies in colon cancer are angiogenesis, sustained proliferative signaling, and immune evasion.

##### Angiogenesis

**Figure 1:**
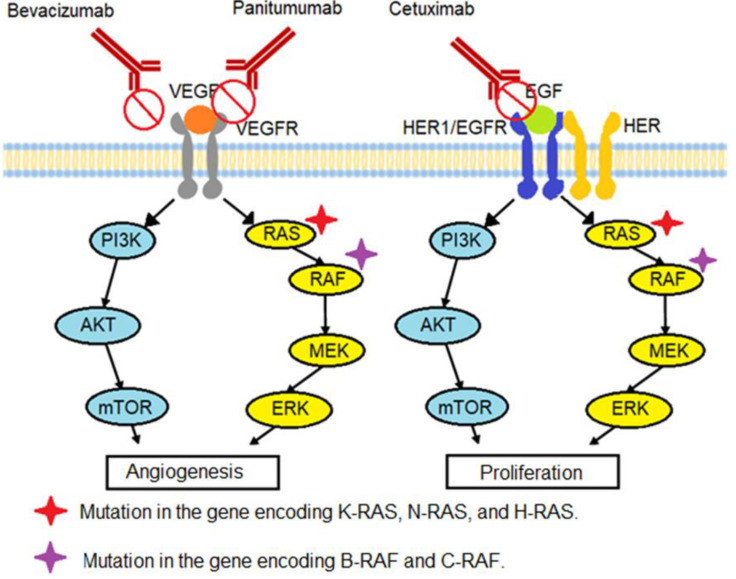
Signaling mechanisms associated with treatment of colon cancer.

Angiogenesis is the formation of blood vessels from existing vessels, which is a key factor in tumor progression [31]. Vascular endothelial growth factor (VEGF) consists of five glycoproteins, VEGF-A, -B, -C, -D, and P1GF, with VEGF-A being the most studied and the target of many antiangiogenic therapies [32]. It binds to receptors known as vascular endothelial growth factor receptors (VEGFRs), which are transmembrane tyrosine kinase receptors, and activates signaling pathways that promote angiogenesis, such as the PI3K–AKT–mTOR pathway (which plays an important role in cell survival and motility) and the KRAS–BRAF–MEK–ERK pathway, as shown in Figure 1. The use of antiangiogenic drugs prolongs the survival of patients by about 20 months, surpassing the 15 months of survival achieved using the FOLFOX or FOLFIRI therapies, which are not significantly different [33,34,35,36,37].

Bevacizumab and panitumumab are the main drugs used to cause an extracellular blockade of these receptors to treat colon cancer.

Bevacizumab: This is a recombinant humanized monoclonal antibody that was approved by the Food and Drug Administration (FDA) in 2004 and the European Medicines Agency (EMA) in 2005 for the treatment of colon cancer [32,38]. It acts by binding to the VEGF-A ligand in the extracellular space, preventing ligand–receptor interactions (Figure 1). This inhibits the proangiogenic pathway that accelerates the growth of tumor cells, thus depriving the tumor cells of essential nutrients and oxygen and promoting their death [39,40,41].

Panitumumab: This is a humanized monoclonal antibody that acts against EGFRs. In 2006, the FDA approved its use as a single agent for the treatment of colon cancer. Today, it is mainly used in combination with other chemotherapeutic agents, as shown in Table 2 [42,43]. Figure 1 illustrates how this molecule binds to the extracellular domain of the EGFR, inhibiting its downstream signaling cascade [42].

##### Sustained Proliferative Signaling

Sustained proliferative signaling is the most studied pathological mechanism of cancer, which is considerably impacted by oncogenes (genes encoding cell proliferation, survival, and growth proteins with one or more mutations) [44]. Approximately 50% of patients with colorectal cancer have mutations in the epidermoid growth factor receptor (HER1, EGFR, or Erbb1) gene. This causes the sustained activation of the PI3K–AKT–mTOR and KRAS–BRAF–MEK–ERK signaling pathways, which promote tumor proliferation, as shown in Figure 1. Cetuximab is one of the anti-EGFR drugs used to treat colorectal cancer [45,46].

Cetuximab: This drug inhibits mitosis in adenocarcinomas by preventing the cell growth so that the cell cannot survive [47].

##### Evasion of Immune System

Tumors have developed several methods of proliferating, including the evasion of the immune system by the binding of receptors and ligands on tumor cells and immune cells. For example, programmed death receptors (PD-1), which are located on the surface of immune cells such as T lymphocytes, bind with the programmed death ligand (PD-L1) on tumor cells to prevent cell death. This phenomenon, along with the activity of another receptor that prevents immune responses, CTLA-4, was described several years ago by James P. Allison and Tasuku Honjo, who consequently received the 2018 Nobel Prize in Medicine and Physiology [48,49,50,51,52].

In recent years, a new type of treatment called immunotherapy has emerged, which uses substances that stimulate or inhibit the immune system to help the body fight cancer, infections, and other diseases [13]. Pembrolizumab is one of the newest immune checkpoint inhibitors (ICIs) included in the NCCN guidelines for the treatment of colon cancer. ICIs alter the tumor microenvironment by favoring the action of the immune system [26].

Pembrolizumab: This is a humanized monoclonal antibody with a high affinity for the human PD-1 receptor, which, when bound to the receptor, blocks its interaction with the PD-L1 and PD-L2 ligands. This increases the efficacy of the T-cell response and the reactivation of antitumor immunity, which reduces tumor growth and prolongs survival. The drug also increases the production of several cytokines, including IL -2, IL -6, IL -7, IFNc, and tumor necrosis factor alpha. This drug should only be used in hot tumors, where the immune system has invaded the tumor and caused inflammation. This is the first oncology drug whose indication is to treat a mutation (microsatellite instability) and not a disease [48,49].

There are some new therapies under study that can improve the survival time of patients, for example, the ICIs such as Avelumab, an anti PD-1 monoclonal antibody that acts like Pembrolizumab. This is being studied in combination with 5-FU and currently shows encouraging results as a treatment for colon cancer [53].

The NCCN guidelines recommend the use of biologics such as bevacizumab, panitumumab, cetuximab, and pembrolizumab in combination with chemotherapy. The main combinations are listed in Table 2 [25,26].

#### 2.3.3. Therapy Safety

The common adverse effects suffered by patients undergoing chemotherapy to treat colon cancer include hair loss (alopecia) [54], sores in the mouth (mucositis) [55], myelosuppression [56], loss of appetite or weight, nausea and vomiting, diarrhea, and nail and skin changes [57,58,59]. Other adverse effects associated with particular drugs include hand-foot syndrome (palmoplantar erythrodysesthesia) [60] and cardiotoxicity caused by 5-FU and capecitabine [61]; neuropathy [62] and allergic reactions caused by oxaliplatin; diarrhea caused by irinotecan, 5-FU, and capecitabine; and mucositis caused by capecitabine, 5-FU, and oxaliplatin [63,64].

## 3. Pharmacogenetics Related to Colon Cancer

Innovative treatments’ aim is to control tumor proliferation mechanisms. Some patients do not respond to colon cancer therapies, and, in many cases, this may be due to pharmacogenetic and pharmacogenomic phenomena. Determining the presence of mutations is key to designing personalized or targeted therapies for each patient, which can improve the effectiveness of colon cancer treatments and the avoidance of unnecessary side effects [65,66].

In the following section, we analyze some genetic variations and their impact on drugs for the efficacy and security of treatment of colon cancer.

### 3.1. K-RAS/N-RAS/H-RAS Gene Mutation

The stimulation of the EGFR activates the RAS–RAF–MEK–ERK signaling pathway, which promotes tumor cell proliferation. Several monoclonal antibodies used to treat colon cancer, such as cetuximab, panitumumab, and bevacizumab, can prevent this by extracellularly inhibiting the activation of this receptor and the RAS–RAF–MEK–ERK pathway, thus stopping cell proliferation. However, patients often have mutations in the genes encoding K-RAS, N-RAS, and H-RAS (44% of metastatic colon cancer cases have somatic mutations in the K-RAS gene). Mutated proteins are active even without EGFR activation. This means that the drugs that block this receptor have little or no clinical efficacy because they block the pathway above the point at which the signals for sustained proliferation are generated, so proliferation continues with the activation of RAF, MEK, and ERK (as shown in Figure 1). Thus, the survival of patients with these mutations is not significantly clinically different when, for example, FOLFIRI monotherapy is used compared with FOLFIRI in combination with cetuximab or FOLFOX monotherapy compared with FOLFOX in combination with panitumumab [64,65,66,67,68,69,70]. In conclusion, anti-EGFR monoclonal antibodies administered as a monotherapy have a substantial clinical effect in colon cancer, improving the survival of patients without K-RAS, N-RAS, or H-RAS mutations, commonly known as wild-type patients [64,65,66,67].

The frequency of RAS mutations is 85% in K-RAS, 15% in N-RAS, and 1% in H-RAS. They are substantially found in codons 12 and 13 of exon 2 (80% are G12D, G12V, G12C, G12A, and G13D) and are consistently lower in codon 61 of exon 3 (5% are Q61H, Q61L, and Q61R) and codon 146 of exon 4 (2% are A146T and A146V) [70].

### 3.2. B-RAF/C-RAF Gene Mutation

Of metastatic colon cancer patients, 10% have mutations in the genes encoding B-RAF and C-RAF. The main mutation (the cause of 90% of RAF mutations) is V600E, wherein the valine at position 600 is replaced by a glutamic acid or glutamate. This somatic mutation in the B-RAF gene generates high proliferation and antiapoptotic behavior in the tumor cell, because, as with the RAS mutation, RAF is continuously activated in the absence of stimuli further up the cascade near EGFR and RAS. This activates the downstream cascade pathway, which then activates MEK and ERK and thus tumor proliferation (Figure 1) [70,71,72,73,74,75].

As with the RAS mutation, an intrinsic resistance to drugs causes inhibition higher up the pathway, which is the case for the anti-EGFR monoclonal antibodies used in colon cancer treatments such as cetuximab, panitumumab, and bevacizumab. Thus, patients with mutations on C-RAF or B-RAF show little or no clinical response to these drugs, with no significant differences in their survival when chemotherapy is used alone or in combination with an anti-EGFR antibody. In conclusion, survival substantially improves only when patients do not have a B-RAF or C-RAF mutation, that is, when they are wild-type [70,71,72,73,74,75,76].

### 3.3. DPDY Genotype

Dihydropyridine dehydrogenase (DPD) is a liver enzyme produced by the DPYD gene that consists of 23 exons on chromosome 1p22. More than 160 single-nucleotide polymorphisms (SNPs) occur in this gene, some of which alter the enzyme’s activity. This enzyme is responsible for inactivating the metabolism of the fluoropyrimidine drugs 5-FU and capecitabine to the metabolite 5-fluoro-5,6-dihydrouracil. Some patients taking these drugs at the usual doses experience toxic effects that can lead to death. Due to variations in the DPYD gene, different DPD phenotypes are expressed in about 5% of patients, who consequently have a DPD deficiency. A single dose may prevent these effects. Genetic testing is important to determine whether patients normally or inadequately express DPD, so that appropriate doses can be calculated to avoid adverse effects, although cost-effectiveness studies and international organizations such as the NCCN recommend this only when serious side effects result from the use of fluoropyrimidines. Variants as IVS+ G > A, I560S, and D949V can lead to this toxic effect [77,78].

### 3.4. Thymidylate Synthase (TS)

**Figure 2:**
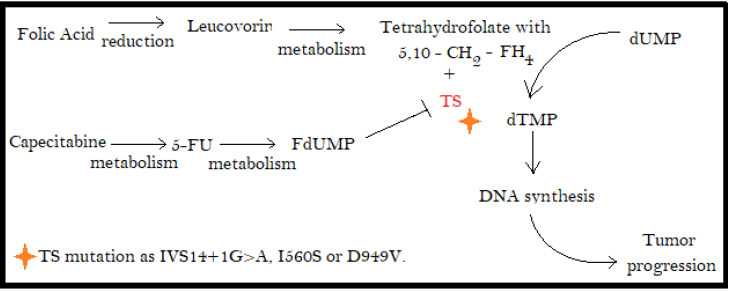
TS mutations and its effect on 5-FU efficacy.

TS is a key enzyme for the 5-FU mechanism of action because it carries a complex formation with fluorodeoxyuridylate (FdUMP) and 5,10-methylenetetrahydrofolate that do not allow the TS to promote the thymidylate (dTMP) formation from uridylate (dUMP). In patients with mutations in the TS gene (TYMS), there are some polymorphisms on TYMS that modulate TS expression and consequently the response of 5-FU against tumoral DNA synthesis, and its toxicity is variable [79,80]. Figure 2 shows the mechanism below. This mutation can be associated with a mutation in an adjacent gene, ENOSF1, that can explain one of the toxicity differences between different 5-FU patient users [81,82].

### 3.5. Methylenetetrahydrofolate Reductase (MTHFR) Gene

MTHFR is an enzyme that allows the change of 5–10-methylenetetrahydrofolate, which is important in the thymidylate formation, to 5- methyltetrahydrofolate. There are some single nucleotide polymorphisms (SNPs) such as A222V than can affect the activity of the MTHFR gene, and this can influence the MTHFR expression that initiates a lower response of this enzyme [83].

### 3.6. ATP Binding Cassette Transporter B (ABCB1 and ABCB2)

Patients with the ABCB1 gene with the I1145I mutation are more prone to suffer diarrhea but have a lower risk of developing a hand-and-food syndrome [79,84].

Other mutations that can affect colon cancer pharmacology therapy may include bilirubin uridine diphosphate glucuronosyl transferase (UGT1A1), excision repair 1, endonuclease non-catalytic subunit (ERCC1), X-ray Repair Cross Complementing 1 (XRCC1), X-ray Repair Cross Complementing 3 (XRCC3), xeroderma pigmentosum group D (XPD), Glutathione S-Transferase Theta 1 (GSTT1), Glutathione S-Transferase Pi 1 (GSTP1), Glutathione S-Transferase Mu 1 (GSTM1), and the Thymidylate synthase enhancer region (TSER) [85,86].

## 4. Conclusions

Currently, medicine offers a promising pathway to personalized therapies. Using point-based analyses enables patients to experience maximum treatment efficacy and safety. Efforts are being devoted to minimizing side effects and improving quality of life and patient survival as much as possible. Current colon cancer therapies are only effective and safe in some patients; for those who have a mutation in certain genes, the therapies fail, and their quality of life deteriorates.

Patients with mutations in the genes coding for RAS and RAF experience a reduction in the effectiveness of therapy because the pathway continues to be active downstream, causing tumor proliferation, despite the blockage caused by the drug. Patients with a mutation in the DPDY gene require a dosage reduction in capecitabine and 5-FU to avoid the occurrence of severe adverse effects. TSYM mutations can modulate down the efficacy of the capecitabine and 5-FU antitumoral response in different patients. When a patient has mutations on the MTHFR gene, it causes a lower response in this enzyme activity. The presence of a mutation on the ABCB1 gene can lead to diarrhea in some patients but minimize the reports of hand-and-foot syndrome. There are other many mutations on genes such as UGT1A1, ERCC1 and Glutathione-Transferase that can clinically impact a lower efficacy for patients.

It is important to address the clinical implication of the oncologist in the study of gene mutations than can influence in the antitumoral response and safety of colon cancer treatments. This tool can impact in a positive way patients’ surveillance of colon cancer because if they use a therapy that is not effective, the cancer will progress, and there are more possibilities to obtain an unwanted response.

Molecular profiles and genetics of patients must be studied to improve treatment decisions. Mutational analyses are used as predictive and prognostic biomarkers for colon cancer treatment, which benefit patient selection and the formulation of personalized treatment strategies that slow disease progression. New treatment guidelines include specific recommendations for treatment based on these genetic mutations.

## Figures and Tables

**Table 1:** Main combinations of chemotherapies used for treatment of metastatic colon cancer [25,26].

Drug Combination	Treatment/Dose	Administration Day	Treatment Cycle
FOLFOX	Oxaliplatin 85 mg/m^2^ intravenous (IV)	1	14 days
	Leucovorin 400 mg/m^2^ IV	1	14 days
	5-FU 400 mg/m^2^ as IV bolus	1	14 days
	5-FU 1200 mg/m^2^/day as continuous IV infusion	2, 3	14 days
CapeOX	Oxaliplatin 130 mg/m^2^ for 2 h	1	21 days
	Capecitabine 1000 mg/m^2^ twice a day orally	1, 2, 3, 4, 5, 6, 7, 8, 9, 10, 11, 12, 13, 14	21 days
FOLFIRI	Irinotecan 180 mg/m^2^ during 30–90 min	1	14 days
	Leucovorin 400 mg/m^2^ IV at the same time as irinotecan	1	14 days
	5-FU 400 mg/m^2^ as IV bolus	1	14 days
	5-FU 1200 mg/m^2^/day as continuous IV infusion	1, 2	14 days

**Table 2:** Main combinations of chemotherapies and biologics used in the treatment of metastatic colon cancer [25,26].

Drug Combination	Treatment/Dose	Administration Day	Treatment Cycle
FOLFOX + Bevacizumab	FOLFOX		
	Bevacizumab 5 mg/kg IV	1	14 days
FOLFOX + Panitumumab *	FOLFOX		
	Panitumumab 6 mg/kg IV for 60 min	1	14 days
FOLFOX + Cetuximab *	FOLFOX		
	Cetuximab 500 mg/m^2^ IV for 2 h	1	14 days
CapeOX + Bevacizumab	CapeOX Bevacizumab 7.5 mg/kg IV	1	21 days
FOLFIRI + Bevacizumab	FOLFIRI Bevacizumab 5 mg/kg IV	1	14 days
FOLFIRI+ Cetuximab *	FOLFIRI Cetuximab 500 mg/m^2^ IV for 2 h	1	14 days
FOLFIRI + Panitumumab *	FOLFIRI Panitumumab 6 mg/kg IV for 60 min	1	14 days

* Only in patients with wild-type KRAS/NRAS/BRAS/BRAF (wild-type: no mutations).

## Data Availability

Not applicable.
